# Biological Reconstruction of Localized Full-Thickness Cartilage Defects of the Knee: A Systematic Review of Level 1 Studies with a Minimum Follow-Up of 5 Years

**DOI:** 10.1177/19476035221129571

**Published:** 2022-10-15

**Authors:** Peter Angele, Johannes Zellner, Steffen Schröter, Johannes Flechtenmacher, Jürgen Fritz, Philipp Niemeyer

**Affiliations:** 1 Sporthopaedicum Regensburg, Regensburg, Germany; 2 Klinik für Unfall- und Wiederherstellungschirurgie, Universitätsklinikum Regensburg, Regensburg, Germany; 3 Abteilung für Unfall- und Wiederherstellungschirurgie, Jung-Stilling Krankenhaus, Diakonie Klinikum GmbH, Siegen, Germany; 4 Ortho-Zentrum, Karlsruhe, Germany; 5 Orthopädisch Chirurgisches Centrum, Tübingen, Germany; 6 OCM—Orthopädische Chirurgie München, München, Germany; 7 Klinik für Orthopädie und Traumatologie, Universitätsklinikum Freiburg, Freiburg, Germany

**Keywords:** knee cartilage defect, autologous chondrocyte implantation, microfracture, osteochondral autograft transplantation, systematic review

## Abstract

**Objective:**

The objective of this study was to evaluate the best available mid- to long-term evidence of surgical procedures for the treatment of localized full-thickness cartilage defects of the knee.

**Design:**

Systematic review using Preferred Reporting Items for Systematic Reviews and Meta-Analyses (PRISMA) guidelines of Level 1 randomized clinical trials (RCTs), meta-analyses of RCTs and systematic reviews with a minimum follow-up of 5 years. Data extracted included patient demographics, defect characteristics, clinical and radiological outcomes, as well as treatment failures.

**Results:**

Six RCTs and 3 Level 1 systematic reviews were included. Two RCTs compared microfracture (MFx) to periosteum-covered autologous chondrocyte implantation (ACI-P), 1 to matrix-associated ACI (M-ACI) and 2 to osteochondral autograft transplantation (OAT). One study compared OAT to collagen membrane covered ACI (ACI-C). The 3 Level 1 systematic reviews/meta-analyses assessed the outcome of MFx, OAT, and various ACI methods in RCTs. OAT showed significantly better outcomes compared with MFx. In the 2 RCTs comparing ACI-P and MFx, no significant differences in clinical outcomes were seen, whereas significantly better outcomes were reported for M-ACI versus MFx in 1 study including patients with larger defects (5 cm^2^), and for ACI-C versus OAT in terms of Cincinnati Score. Higher failure rates were reported for MFx compared with OAT and for OAT compared with ACI-C, while no significant differences in failure rates were observed for ACI-P compared to MFx.

**Conclusion:**

Restorative cartilage procedures (ACI-C or M-ACI and OAT) are associated with better long-term clinical outcomes including lower complication and failure rates when compared with reparative techniques (MFx). Among the restorative procedures, OAT seems to be inferior to ACI especially in larger defects after longer follow-up periods.

**Level of evidence:**

Level I: Systematic review of Level I studies

## Introduction

Chondral or osteochondral lesions induce pre-arthritis, a stage where cellular processes influenced by risk factors are initiated but have not yet resulted in macroscopic structural changes of osteoarthritis (OA). Similar to meniscus injuries, the stress effect of cartilage damage increases with lesion size while the compensatory ability of the surrounding intact joint surface decreases. This is why lesions larger than approximately 2 cm^2^ are at significant risk of arthritis in the spontaneous course or after unsuitable defect treatment compared with smaller lesions.^1-4^

Once pathologic cartilage or osteochondral damage has occurred due to an acute event or recurring microtrauma, the destruction of the joint can continue to progress due to poor joint biomechanics. This leads to recurring inflammatory episodes accompanied by the induction of cartilage-destructive metabolic pathways.^4,5^

Chronic exposure of the affected joint to high or peak loads, which may result, inter alia, from obesity or high-impact sports activity, can significantly accelerate the course of the disease. Therefore, in addition to the extent of the joint deformity causing pre-arthritis (e.g., the extent of primary cartilage damage, axis deviation, instability, meniscus damage) and the accompanying risk factors (e.g., obesity), time is also an essential factor in the development of OA.^5,6^

The relationships described above indicate what is essential from a medical point of view. As much as possible, the therapeutic goal must be the prevention or minimization of pre-arthritis and related risk factors to prevent or at a minimum delay the development of degenerative changes.

Most comprehensive studies show that the clinical outcome success of cartilage repair surgery declines with longer symptom duration and the number of previously failed interventions.^7-10^ Therefore, repair of localized cartilage defects with persisting symptoms and in the absence of contraindications should be carried out as early as possible and with a procedure suitable for the defect.

Furthermore, in addition to the repair of localized full-thickness cartilage defects, which are often associated with a significant reduction in quality of life similar to manifest gonarthritis, concomitant pathologies such as axis malalignment or joint instability must also be addressed independently of the cartilage repair modality used.^4,11,12^

### Surgical Techniques to Treat Cartilage Defects

There is no standard operative treatment suitable for all shapes, sizes, or locations of focal chondral or osteochondral knee lesions from a scientific or routine clinical care point of view. The currently available clinical options complement each other in terms of their indication and have recently been differentiated into those with reparative or restorative properties.

Reparative methods (such as bone marrow-stimulating techniques with and without biomaterial augmentation) are characterized by the formation of fibrous cartilage, whereas restorative methods such as autologous (OAT) or allogeneic osteochondral transfer (OCA) and autologous chondrocyte implantation (ACI) form cartilage with hyaline properties.^13^

There is increasing evidence from various studies with longer follow-up periods that restorative procedures have better long-term results with lower failure rates.^3,14-17^

The aim of this systematic review was to identify the most appropriate surgical therapy for patients with cartilage defects of the knee based on the best available evidence. Therefore, only Level 1 randomized controlled trials (RCTs), meta-analyses, and systematic reviews with a follow-up time of at least 5 years were included in the systematic review.

## Methods

### Search Strategy

The systematic literature review was performed in accordance with the Preferred Reporting Items for Systematic Reviews and Meta-Analyses (PRISMA) guidelines^18^ using the PubMed database. All publications retrieved were selected according to predefined inclusion/exclusion criteria described in the titles/abstracts and/or full-text. The search strategy, keywords used and filters applied in the search are reported in Supplementary Appendix 1.

### Selection Criteria

Criteria for selection and further evaluation were full-text articles in English or German with a publication date between 2011 and 2021, clinical (human) data, evidence Level 1 RCTs, meta-analyses of RCTs and systematic reviews, comparison of at least 2 cartilage repair methods with at least 20 participants per treatment arm in RCTs, defect localization/treatment in the knee joint and reported clinical outcome after a follow-up of at least 5 years. Treatment of OA was an exclusion criterion as the defect situation in OA is different from non-OA knees. Articles were screened by 2 separate investigators (P.A. and J.Z.) and discrepancies in ratings or disagreements were resolved by discussion and consensus with the third author (P.N.).

The risk of bias within each study was evaluated in accordance with the methods of the Cochrane Collaboration tool.^19^

### Data Extraction

Data extracted from the selected articles included patient demographics, previous knee surgery, duration of symptoms, articular cartilage defect size, surgical technique, clinical outcome measures, radiological assessment, and treatment failures. Primary outcome measures were validated clinical scores (e.g., Knee Injury and Osteoarthritis Outcome Score [KOOS] or International Knee Documentation Committee [IKDC]). Secondary outcome measures included failure rates and radiological outcome. Headline results of the individual studies were summarized and discussed according to the treatment techniques they compared (e.g., microfracture versus cartilage restorative techniques and other comparative studies).

## Results

### Overview of Literature Search Output

**Figure 1.**
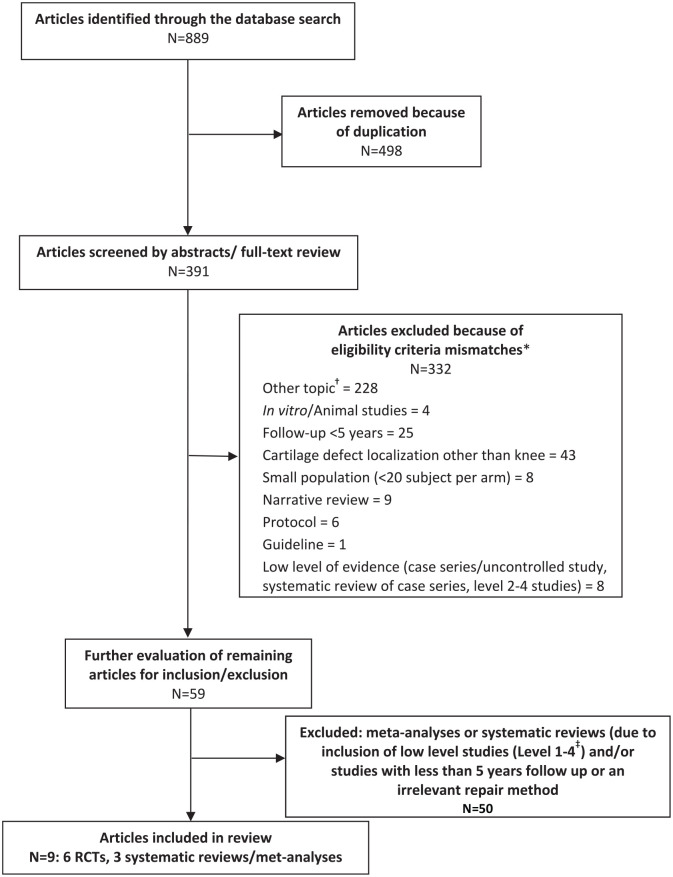
PRISMA flow chart of the systematic literature search. *Publications may not match one or several inclusion/exclusion criteria. However, each excluded publication is counted only once. ^†^Other topic includes: no cartilage defect or cartilage repair at all, treatment with experimental methods like mesenchymal stem cells, injection of different substances, treatment with physical therapy, treatment of osteoarthritis, meniscus transplantation, and methods of rehabilitation. ^‡^The level of evidence was considered as reported in the respective publications; however, if no level of evidence was given in publications, the following definition was used adapted from the definitions of the Canadian Task Force and the Centre for Evidence-Based Medicine described in Burns *et al*.^20^ (see also https://www.cebm.ox.ac.uk/resources/levels-of-evidence/oxford-centre-for-evidence-based-medicine-levels-of-evidence-march-2009): Level 1 = individual randomized controlled trial and systematic reviews/meta-analyses of randomized controlled trials; Level 2: systematic review of cohort studies, cohort studies, low quality RCTs; Level 3: systematic review of case-control studies, case-control studies; Level 4: case series, poor quality cohort and case-control studies; Level 5: expert opinion.

A total of 889 records from the PubMed database were identified (**Fig. 1**). Of those 889 publications, 498 duplicate publications were removed and the remaining 391 publications were screened on the basis of abstract/full text review. The reviewer excluded 332 records based on the predefined exclusion/inclusion criteria.

Most articles were excluded under an “other topic” category which included no cartilage defect or no cartilage repair at all, treatment with methods like mesenchymal stem cells with or without additional microfracture, bone marrow concentrate as cell source, injection of different substances, treatment with physical therapy, methods of rehabilitation, and OA treatment.

Of note, most of the excluded publications met several exclusion criteria. For example, studies on mesenchymal stem cell therapies or other treatments were usually small (<20 patients) and no Level 1 trials. Other more frequent reasons for exclusion were defect localization other than knee joint (n = 43) or follow-up less than 5 years (n = 25).

A total of 59 unique publications (including systematic reviews/meta-analyses) were selected for further evaluation by intensive full-text review. Of these, 50 publications were excluded based on the defined inclusion/exclusion criteria, leaving a total of 9 articles for inclusion: 6 RCTs and 3 systematic reviews/meta-analyses (**Fig. 1**).

Two of the included articles were RCTs that compared first-generation ACI using a periosteal patch (ACI-P) to microfracture (MFx),^21,22^ 1 study compared third-generation matrix-associated ACI (M-ACI) to MFx,^23^ and 2 studies evaluated osteochondral autograft transplantation (OAT) versus microfracture.^24,25^ A comparison of OAT and second-generation, collagen membrane covered ACI (ACI-C) was covered in another study.^26^ The 3 Level 1 systematic reviews/meta-analyses assessed the outcome of different cartilage repair procedures, that is, MFx, OAT, and various ACI methods, in randomized controlled trials.^27-29^ No RCTs fulfilling the inclusion criteria for the current review were available on cartilage repair by means of bone marrow concentrate, mesenchymal stem cells, particulated juvenile allograft cartilage, or autologous minced cartilage.

For practicability reasons, the results described here refer to the 6 RCTs only. The systematic reviews/meta-analyses are referred to in the discussion section to support the conclusions drawn from the RCTs.

### Patient Demographics and Baseline Characteristics

The 6 RCT articles included in this systematic review comprised 520 patients with a mean age ranging from 24.3 to 35.0 years. Follow-up periods ranged from 5 years up to a mean of 16 years. The duration of symptoms prior to cartilage repair varied widely from 2.6 months to 7.2 years. Mean defect sizes ranged from a mean of 2.4 to 5.1 cm^2^. Trial and patient characteristics are summarized in **Table 1**.

**Table 1.** Trial Characteristics of the Included Studies.

Study	Treatment	Patients (n)	Patients Completed Follow-Up (n)	Follow-Up (Years)	Mean Age (Years)	Mean Defect Size (cm^2^)	Mean Duration of Symptoms (Months)	Mean Prior Surgeries (n)
Brittberg *et al*.^23^	M-ACI	65	65	5	35^a^	5.1	—	—
	MFx	63	59		34^a^	4.9		
Knutsen *et al*.^21^	ACI-P	40	40	15	33.3	5.1	36	1.6
	MFx	40	38		31.1	4.5		1.4
Vanlauwe *et al*.^22^	ACI-P	51	43	5	33.9^a^	2.6 ^a^	1.97 years^a^	77%^a^
	MFx	61	55		33.9^a^	2.4 ^a^	1.57 years^a^	88%^a^
Solheim *et al*.^24^	OAT	20	—	16 (15-17)	31	3.4	52	—
	MFx	20			35	3.6	58	
Gudas *et al*.^25^	OAT	30	28	10.4 (9-11)	24.3	2.80	21.3	—
	MFx	30	29			2.77		
Bentley *et al*.^26^	ACI-C	58	53	min. 10 (10-12)	30.9	4.4 (1-10.5)	7.2 years	1.5
	OAT	42	41		31.6	4.0 (1-20)		

MFx = microfracture; OAT = osteochondral autograft transplantation; ACI-P = periosteal autologous chondrocyte implantation; ACI-C = collagen-covered autologous chondrocyte implantation; M-ACI = matrix-associated autologous chondrocyte implantation; m﻿in =﻿ minimum.

a Numbers are given by Saris *et al*.^30^

### Risk of Bias

**Figure 2.**
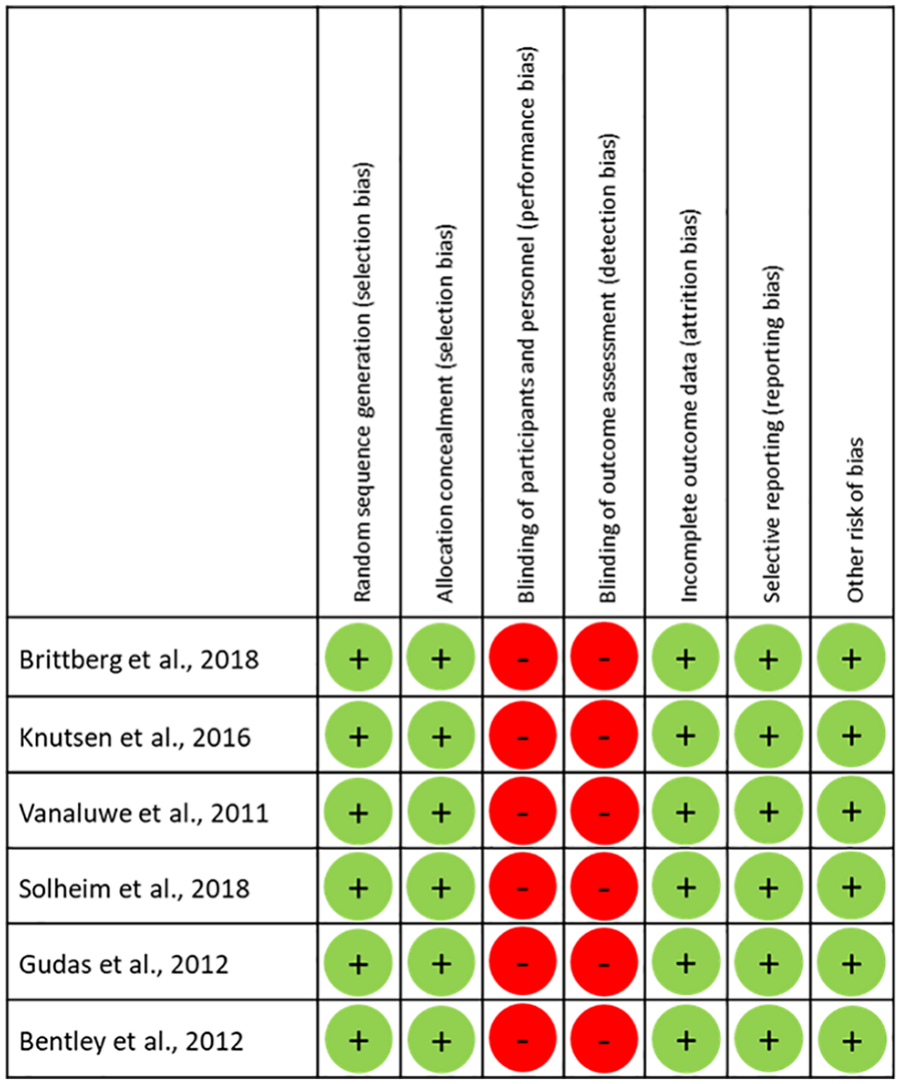
Results of risk of bias assessment for individual trials. + = no bias assumed; – = bias cannot be excluded.

**Figure 2** shows the potential risk of bias for the individual studies. All of the included studies presented Level I evidence, with a low risk of selection, attrition, reporting, or other biases. However, performance bias was potentially present in all included studies, due to the non-blinded nature of the various surgical techniques.

Furthermore, it was not possible to blind the patients to their interventions in studies that involved the use of 2-stage interventions (ACI) and 1-stage procedures (MFx or OAT). In 3 of 6 studies, the outcome assessors were not blinded to the study treatment,^21,23,26^ in 1 study a blinded unbiased observer performed preoperative and follow-up examinations^25^ and for the 2 remaining studies no information on assessor blinding was available. However, a potential detection bias could not be excluded as patient-reported outcome measures were used in all these non-blinded studies.

### Clinical Scores

The results of the treatment group comparisons in clinical scores are summarized (**Table 2**).

**Table 2.** Clinical Outcome in the Included Studies.

Study	KOOS	IKDC	ICRS	Tegner	Lysholm	SF-36		EQ-5D-VAS	VAS Pain	Cincinnati	Stanmore-Bentley Score
						Physical	Mental				
MFx vs. ACI											
Brittberg *et al*.^23^	↑ M-ACI^a^	NS				↑ M-ACI	NS	↑ M-ACI		↑ M-ACI	
Knutsen *et al*.^21^				NS	NS	NS			NS		
Vanlauwe *et al*.^22^	NS^b^										
MFx vs. OAT											
Solheim *et al*.^24^					↑ OAT						
Gudas *et al*.^25^			↑ OAT	↑ OAT							
ACI vs. OAT											
Bentley *et al*.^26^										↑ ACI-C	NS

NS = no significant difference; ICRS = International Cartilage Repair Society; KOOS = Knee Injury and Osteoarthritis Outcome Score; SF-36 = Short Form 36; IKDC = International Knee Documentation Committee; EQ 5D = European Quality of Life; VAS = visual analog scale; MFx = microfracture; OAT = osteochondral autograft transplantation; ACI = autologous chondrocyte implantation; ACI-C = collagen-covered autologous chondrocyte implantation; M-ACI = matrix-associated autologous chondrocyte implantation.

a Significant difference in co-primary endpoint of KOOS pain and function and activities of daily living.

b For patients with symptom onset less than 3 years, the overall KOOS showed a statistically significant and clinically relevant difference in favor of the ACI-P group.

In the 2 RCTs comparing MFx and OAT, OAT showed significantly better outcomes in the ICRS and Tegner scores after 10 years^25^ as well as the Lysholm score after 16 years.^24^ Both trials included patients with comparatively small cartilage defects (mean 2.8-3.6 cm^2^).

Two RCTs compared MFx and ACI-P.^21,22^ Patients in the study conducted by Vanlauwe *et al*.^22^ had comparatively small defects (mean size of approximately 2.5 cm^2^), while patients in the trial reported by Knutsen *et al*.^21^ had larger defects (mean defect sizes of 4.5 and 5.1 cm^2^ for MFx and ACI-P, respectively). There were no significant differences between repair modalities in overall KOOS after 5 years^22^ or Tegner, Lysholm, SF-36, and visual analog scale (VAS) pain after 15 years.^21^

However, Vanlauwe *et al*.^22^ reported a statistically significant and clinically relevant difference in overall KOOS improvement in the ACI group over the MFx group at 60 months in a subgroup of patients with symptom onset less than 3 years (*P* = 0.026). Significant differences versus MFx were also observed in the pain and quality of life domains in this patient subgroup.

In contrast, significantly better outcomes for third-generation M-ACI compared with MFx have been reported by Brittberg *et al*. in patients with larger defects of approximately 5 cm^2^.^23^ In this study, M-ACI was shown to be superior to microfracture in the co-primary endpoint of improvement in KOOS pain and function after 5 years (*P* = 0.022) and also in the activities of daily living subscore (*P* = 0.007).

Furthermore, in the study by Bentley *et al*., ACI-C was shown to be superior to OAT in terms of the Cincinnati Score while no differences were observed in the Stanmore-Bentley score after a minimum follow-up of 10 years.^26^ Mean defect sizes in this study were 4.4 and 4.0 cm^2^, respectively.

### Failure Rates

The majority of studies defined failure as a need for reoperation.^21-23,25,26^ Additional criteria for graft failure such as clinically poor results with arthroscopic evidence of failure or <10% improvement in the KOOS pain subscale were also included in the failure definition of 2 studies.^23,26^ Failure definitions and failure rates are summarized in **Table 3**.

**Table 3.** Failure Rates for Different Cartilage Repair Techniques in the Included Studies.

Study	Follow-Up (Years)	Treatment	Failure	Definition of Failure	Significant Difference
MFx vs. ACI					
Brittberg *et al*.^23^	5	M-ACI	1	Patient and physician global assessment result the same or worse than at baseline after week 24 <10% improvement in the KOOS pain subscale Surgical retreatment necessary	NS
		MFx	3		
Knutsen *et al*.^21^	15	ACI-P	17 (42.5%)	Reoperation because of symptoms resulting from a lack of healing of the treated defect. The need for shaving or trimming of a lesion was not considered a failure.	NS
		MFx	13 (32.5%)		
Vanlauwe *et al*.^22^	5	ACI-P	7 (13.7%)	Re-intervention affecting more than 20% of the graft area	NS (*P* = 0.561)
		MFx	10 (16.4%)		
MFx vs. OAT					
Solheim *et al*.^24^	16	OAT	1 (5%)	Only knee replacement procedures reported	-
		MFx	3 (15%)		
Gudas *et al*.^25^	10.4	OAT	7 (14%)	Reoperation due to symptoms to the primary defect	*P* < 0.05
		MFx	11 (38%)		
ACI vs. OAT					
Bentley *et al*.^26^	min. 10	ACI-C	10 of 58 (17%)	Clinically poor results with arthroscopic evidence of failure of the graft or revision surgery to the defect of any kind	*P* < 0.001
		OAT	23 of 42 (55%)		

MFx = microfracture; OAT = osteochondral autograft transplantation; ACI-P = periosteal autologous chondrocyte implantation; ACI-C = collagen-covered autologous chondrocyte implantation; M-ACI = matrix-associated autologous chondrocyte implantation; NS = no significant difference; m﻿in =﻿ minimum.

Bentley *et al*.^26^ reported significantly less failures with ACI-C compared with OAT at a minimum of 10-year follow-up, 17% and 55%, respectively (*P* = 0.001), using a Kaplan-Meier worst case scenario comparison in which patients lost to follow-up were assumed to have been failures (5 of 58 in the ACI-C group and 1 of 42 in the OAT group). Gudas *et al*.^25^ identified a significantly greater failure rate with microfracture (38%) compared with OAT (14%) (*P* < 0.05) at 10 years. Accordingly, Solheim *et al*.^24^ reported more knee replacements with MFx (3 patients, 15%) than with OAT (1 patient, 5%) after 16 years, although the numbers were too low to draw any definitive conclusions (other treatment failures were not addressed in this article).

Comparing first-generation ACI-P and MFx, differences in failure rates were not statistically significant in the studies by Vanlauwe *et al*.^22^ and Knutsen *et al*.^21^ after 5 and 15 years, respectively. However, failure rates in the study by Knutsen *et al*.^21^ were nominally higher for ACI-P after 15 years (42.5% vs. 32.5%).

Very few treatment failures were observed in the study by Brittberg *et al*.^23^ with only 1 failure in the M-ACI group and 3 failures in the MFx group during the follow-up period of 5 years.

### Radiological Outcomes

Radiological outcomes of the studies included in our review are summarized in **Table 4**. No patients showed evidence of OA at 3 years post-surgery in the cohort of patients reported by Gudas *et al*. However, at 10 years 48% of patients in the MFx group and 25% in the OAT group had developed degenerative changes, but these differences were not significant (*P* = 0.83).^21^ In the same study, significantly better Magnetic Resonance Observation of Cartilage Repair Tissue (MOCART) subscore results were observed with OAT for defect filling, integration to border zone, surface of repair tissue, subchondral lamina and bone as well as adhesions.

**Table 4.** Imaging Outcomes for Different Cartilage Repair Techniques in the Included Studies.

Study	Treatment	Imaging	Outcome	Significant Difference
MFx vs. ACI				
Brittberg *et al*.^23^	M-ACI vs. MFx	MRI in 120 patients at year 5	Improvement in defect filling in both treatment groups	No difference
Knutsen *et al*.^21^	ACI-P vs. MFx	OA was assessed defined as Kellgren and Lawrence grade ≥2	ACI-P: 57% had signs of OA MFx: 48% had signs of OA	NS
Vanlauwe *et al*.^22^	ACI-P vs. MFx	OA was assessed defined as Kellgren and Lawrence grade ≥2	4 of 49 patients (8%) had a Kellgren grade 2 score; there was no significant difference in the frequency of radiographic changes between treatment groups	NS
MFx vs. OAT				
Gudas *et al*.^25^	OAT vs. MFx	Assessment of MOCART score and Kellgren and Lawrence grade	MRI after 10 year performed in 17 MFx and 12 OAT patients. Significantly better MOCART subscore results with OAT for: • Defect filling • Integration to border zone • Surface of repair tissue • Subchondral lamina • Subchondral bone • Adhesions	*P* < 0.05
Kellgren and Lawrence grade: 7 (25%) in the OAT and 14 (48%) in the MFx group had evidence of Kellgren and Lawrence grade I OA	NS (*P* = 0.083)			

MFx = microfracture; OAT = osteochondral autograft transplantation; ACI-P = periosteal autologous chondrocyte implantation; M-ACI = matrix-associated autologous chondrocyte implantation; NS = no significant difference; OA = osteoarthritis; MOCART = Magnetic Resonance Observation of Cartilage Repair Tissue.

Two studies compared ACI-P and MFx with regard to development of osteoarthritic changes over time. Vanlauwe *et al*. reported osteoarthritic changes in 8% of patients overall at the 5-year follow-up and no differences between the ACI-P or MFx treatment techniques.^22^ At a mean of 15 years postoperative, 57% of ACI-P patients and 48% of MFx patients had developed signs of OA in the study conducted by Knutsen *et al*.^21^ This difference however was not statistically significant.

In a study by Brittberg *et al*.,^23^ magnetic resonance imaging (MRI) evaluation of structural repair was performed in 120 patients at year 5 in patients treated with either M-ACI or MFx. The MRI evaluation showed improvement in defect filling for both treatments; however, no statistically significant differences were noted between treatment groups.

## Discussion

Only 9 studies at the highest level of evidence with a follow-up of at least 5 years could be identified in the field of knee cartilage repair and therefore the following discussion will also take into account data from additional clinical studies on the respective techniques. In 5 of 6 RCTs included in our review, microfracture was used as comparator and is discussed only in this context rather than separately as a repair option. No RCTs meeting the inclusion criteria for the current review were available on cartilage repair by means of bone marrow concentrate, mesenchymal stem cells, particulated juvenile allograft cartilage, or autologous minced cartilage.

### Autologous Chondrocyte Implantation Versus Microfracture

A long history of ACI products documented in a comprehensive series of published systematic reviews, meta-analyses, and clinical guidelines has demonstrated that ACI, and in particular M-ACI, is superior to MFx, particularly in larger lesions and in long-term clinical outcomes.^16,27,29,31-35^

In the randomized Level 1 phase III study on M-ACI versus MFx for the treatment of large cartilage defects (5 cm^2^) included in our review, significantly better clinical results were reported for M-ACI after 2 and 5 years.^23,36^ Similar results were reported by Basad *et al*.^37^ for defect sizes of 4 to 10 cm^2^. The superior efficacy of M-ACI in the treatment of larger defects (3-20 cm^2^) was also confirmed in a predictor analysis in one trial where a defect size >4 cm^2^ was predictive for better outcome with M-ACI.^36^

In a phase III clinical trial including a population with smaller defect size (1-4 cm^2^), non-inferiority, but not superiority, of the M-ACI product to MFx was demonstrated at 24 months.^38^ Similar results were also found in other controlled clinical studies with a M-ACI product in smaller defects.^30,39,40^ Only 1 Phase II RCT has demonstrated significant clinical benefits for M-ACI compared with MFx after 24 months in smaller defects.^41^ However, the small sample size with 21 patients in the M-ACI group and 9 patients in the MFx group limits the interpretation of these results. In a Level 1 RCT including 35 patients (18 M-ACI and 17 MFx), M-ACI had better structural outcomes than those who underwent MFx in chondral lesion with a mean size of 1.8 cm^2^ at 1 to 6 years postoperatively. In the same study, both groups of patients showed significant clinical improvements at final follow-up compared with their preoperative status but M-ACI showed significant superiority at 4 years for the majority of the KOOS subscales and for the Tegner scale at 4 to 6 years. The responder rates at 6 years were 53% and 77% for MFx and M-ACI, respectively.^42^

Long-term maintenance of significant improvements at up to 5 years and for more than 5 and 10 years after M-ACI/ACI has been reported in several clinical studies^17,40,43-55^ and reviews.^28,56^ While MFx is still considered a treatment option for smaller defects (<2-2.5 cm^2^), there is evidence from several clinical studies, systematic reviews, and meta-analyses that even with smaller and medium defect sizes, MFx loses its clinical efficacy over time.

A systematic review by Na and co-workers showed that significant clinical improvement was achieved after 5 or more years with both ACI and MFx, but the results after ACI-C and M-ACI were significantly better compared with MFx as determined by the KOOS ADL assessment, Tegner Activity Scale score, and IKDC objective and subjective scores.^57^ This review found no significant difference in the treatment failure rate between these 2 methods. The review is in agreement with previous findings from a systematic review of 20 clinical studies (1,469 athletes) showing that patients treated with ACI reached the highest activity level when compared with other cartilage repair techniques, including MFx or mosaicplasty. A durability of up to 96% after ACI was observed as late as 9 years after surgery even under the high physical demands of professional football.^58^ In a recently published randomized controlled trial (Level 1), 93.3% of patients were satisfied with M-ACI for relieving their pain at 10 years, with 83.3% satisfied with their ability to participate in sports and, therefore, the authors concluded that M-ACI provided high satisfaction levels and tissue durability beyond 10 years.^55^ In contrast, 1 MFx study demonstrated that the rate of return-to-sports decreased from 80% at 2 years to 55% by the 6-year time point.^59^ A systematic review showed a similar decline of functional knee results after MFx during a follow-up of 2 to 5 years.^60^ In another prospective study, nearly half of the patients (50 of 110) treated with MFx had a poor outcome (knee replacement or Lysholm score below 64) at a median follow-up of 12 years, and 43 of 110 patients had 1 to 4 additional surgeries, including 7 knee replacements.^61^

In a meta-analysis including 3,894 patients comparing short- (1-4 years), medium- (5-9 years), and long-term (≥10 years) results of MFx, OAT, and ACI/M-ACI, best results concerning pain relief within the first 4 years were observed for MFx. However, and in contrast to ACI, MFx lost its ability of clinically relevant pain relief during mid-term follow-up, whereas even in the long-term, this was not the case with ACI.^62^ In this context, it should also be mentioned that persisting knee pain is considered to be a predictor for OA and an eventual joint prosthesis.^63,64^ Against the background of the described relation between chronic pain and the development and progression of degenerative joint alterations, it is understandable that increasing rates of MFx treatment failures and OA regardless of lesion size beyond 5 years was a common observation in other studies.^16,65^ In our systematic review, Gudas *et al*.^25^ observed degenerative changes in 48% of patients in the MFx group (25% in the OAT group) and a significantly greater failure rate of 38% with MFx compared with OAT (14%) at 10 years. At a mean of 15 years of follow-up, 48% of MFx patients and 57% of ACI-P patients had developed signs of OA in the study conducted by Knutsen *et al*.^21^

Similar results have been reported in a Level 1 network meta-analysis and in another systematic review where significantly higher revision and failure rates were found at long-term follow-up for MFx and osteochondral autograft/mosaicplasty when compared with ACI-C/M-ACI.^27,29^ Up to 5 years however, no significant differences in failure rates between ACI and MFx were reported^29,57,66,67^ and also in the 2 studies with a follow-up of 5 years included in our systematic review, no differences in failure rates were observed between ACI and MFx.^22,23^ For ACI-P however, despite a longer follow-up of 15 years, no differences in failure rates compared with MFx were reported.^21^

Together, these are the main reasons why many authors now recommend longer follow-up periods for cartilage clinical trials, as the therapeutic goal is not only the relief of acute pain and discomfort, but also the achievement of durable long-term results and the potential to prevent or at least delay the early onset of OA and knee arthroplasty. It is widely accepted that focal cartilage defects of the knee are a risk factor for the development of OA, and early joint replacement, which is increasingly discussed controversially, should be avoided as much as possible.^1,3,4,68-73^ The improved long-term durability of M-ACI is why it is now considered economical despite the initially higher costs of 2 surgical interventions and the necessary cell cultivation.^74,75^

On the other hand, ACI-P hardly plays any role in clinical practice anymore due to its higher surgical comorbidity and complication rates, such as periosteal hypertrophy.^4,75^ Furthermore, the results of a meta-analysis performed by Deng *et al*.^76^ indicated that M-ACI had significant better efficacy than ACI-P did. Accordingly, no difference was observed in the 2 studies investigating ACI-P versus MFx in our systematic review^21,22^ (irrespective of defect size), while M-ACI has shown superior efficacy over MFx in various clinical scores as reported by Brittberg *et al*.^23^ or recently by Dhillon *et al*.^77^ (systematic review of Levels I-II trials).

It is also worth mentioning that patients undergoing ACI as second-line treatment after previously failed MFx have a significantly higher risk of treatment failure and worse subjective outcomes compared with patients undergoing primary ACI.^10,78^ With respect to the described differences in mid- to long-term results of cartilage repair methods, these findings are an important consideration when discussing postoperative expectations with surgical candidates.^62^

The reason underlying the inferior long-term results for MFx when compared with ACI is thought to relate to the poor tissue quality and degree of defect fill particularly in larger lesions.^4,79^ However, Brittberg *et al*.^23^ (as presented in this systematic review) did not observe any differences in defect filling between M-ACI and MFx after 5 years (mean defect size 5 cm^2^). In this context, it has been shown that filling of smaller, well-shouldered cartilage defects even with non-hyaline repair tissue still improves short-term function, while the histological repair tissue quality becomes more important long term for larger defects.^27,29,60,62^ In a meta-analysis, MFx was found to produce primarily fibrocartilage repair tissue that matures differently from ACI tissue.^80^ ACI repair tissue matures to become more hyaline-like (a process that takes up to 5 years)^81^ with increased stiffness, while the fibrocartilage formed after MFx can enlarge over time but lacks the maturation into cartilage with hyaline properties.

The formation of intralesional osteophytes via endochondral ossification (a process in which cartilaginous tissue is gradually replaced by bone) is another complication associated with bone marrow stimulation techniques. This process is accompanied by disruption, vascularization, elevation, and sclerosis of the subchondral bone as well as cyst formation resulting in a progressive thinning and degeneration of the covering cartilage over time, which probably explains the improved durability of ACI compared with MFx.^82,83^

Several studies have shown that adverse bone formation occurs in up to 70% of all lesions treated by MFx and peaks between 4 and 5 years after treatment.^82,84^ In a prospective study on MFx, subchondral bone overgrowth was observed in over 90% of treatment failure patients, with a risk of failure 10 times higher than in patients who showed no osseous overgrowth (*P* < 0.01).^85^ Among the examined procedures (MFx, OAT, OCA, first-generation ACI) for the surgical treatment of cartilage defects of the knee covering a total of 47,207 cases from a large U.S. commercial insurance database, MFx presented the greatest risk of eventual conversion to total knee replacement (*P* < 0.0001).^86^ Taken together, these results are in line with a recently published Level 1 meta-analysis by Zamborsky and Danisovic^28^ of RCTs that ranked MFx as the procedure with the worst long-term clinical outcome compared with ACI/M-ACI and OAT.

### Osteochondral Transfer Versus Microfracture or Autologous Chondrocyte Implantation

The 2 studies comparing OAT with MFx included in our systematic review with follow-up times of up to 17 years showed significantly better clinical results and lower failure rates for mosaicplasty in comparison with MFx for the treatment of chondral and osteochondral defects. One limitation of these studies is the smaller mean defect size (2.8 and 3.5 cm^2^).^24,25^ Similar data on the comparison of OAT with MFx were published in a systematic review including 9 clinical studies with Level 1 or 2 evidence.^87^

The prospective randomized Level 1 study by Bentley *et al*.^26^ on mosaicplasty versus ACI included in this review showed significantly better results and lower failure rates (*P* < 0.001) for ACI-C over the 10-year study period (average defect size of approximately 4 cm^2^ in both groups). Another more recent study on the long-term comparison of mosaicplasty and M-ACI showed significantly lower failure rates for M-ACI over the 12-year period (*P* = 0.016) in smaller lesions (2 cm^2^ average). Patients with defects larger than 2 cm^2^ that received mosaicplasty performed significantly worse in the Tegner Activity and objective IKDC scores than patients with smaller lesions, which was not the case in the M-ACI group.^88^

In 2 systematic reviews at the highest level of evidence, significantly higher complication rates (including treatment failure) and reoperation rates were found for mosaicplasty long term (>10 years) compared with ACI/M-ACI.^27,29^

Overall, OAT for smaller lesions of the femoral condyles generally leads to good clinical results even after longer periods of time and with the shortest rehabilitation phase compared with other methods, especially in high-impact sports.^13,84,89,90^ However, increasing complication and failure rates have been reported for larger defects requiring the use of more than 2 cartilage-bone cylinders.^74,87,91^ Failure to achieve ideal joint surface congruence, similar to an intra-articular fracture that heals with step formation, results in abnormal loads and hence in degenerative changes of the corresponding cartilage surface.^29,92^

OAT has also proven to be problematic for various reasons in the treatment of patella defects and the tibial articular surfaces.^93-95^ Methods such as mosaicplasty are therefore mainly recommended for the treatment of osteochondral defects of the femoral condyles that do not exceed a size of 2 to 3 cm^2^.^4,87,89,91^

Allogeneic cartilage-bone transfer has a wide range of indications and is often used in the United States as a salvage procedure for large osteochondral defects.^74^ Early revisions are not uncommon as described in a systematic review showing an allograft failure rate of 18% and a reoperation rate of over 30% at a mean follow-up of 8.7 years.^95^ A recently published retrospective study including 82 patients with ACI and 66 with osteochondral allograft transplantation (mean follow-up of 6.7 years) indicated that both techniques provided similar patient-reported outcomes with or without concomitant procedures for the treatment of symptomatic knee cartilage defects. However, the overall rate of failure, defined as graft failure with revision surgery and/or conversion to arthroplasty, was significantly greater in the allograft group (21% vs. 4%; *P* = 0.002), particularly for multifocal and condylar lesions.^96^

The use of suitable allografts is limited by their limited availability (particularly in Europe) and the risk of disease transmission. Due to the invasiveness of the procedure, especially in the case of large lesions, the options for revision after allograft failure are limited.^97^

### Bone Marrow Concentrate, Mesenchymal Stem Cells, Particulated Juvenile Allograft Cartilage, and Minced Cartilage

As outlined in the method section, studies on techniques mentioned in the heading were usually small (<20 patients), with no Level 1 trials, or studies with a follow-up less than 5 years. Therefore, it is still unclear whether they are superior to bone marrow stimulation techniques in terms of their effectiveness and whether they can achieve similarly good results as ACI/M-ACI, especially with regard to larger defects and longer follow-up times.^98^ As for all other cartilage repair procedures, results from prospective, high-quality studies with defined target indications and longer follow-up times are required, before they or other relatively new methods can also be subject to an evidence-based assessment and as a result may or may not be included in a corresponding treatment algorithm.^28^

## Conclusion

Different surgical procedures are available for the biologic reconstruction of localized full-thickness cartilage defects of the knee. Our systematic review including RCTs on the highest level of evidence with a minimum of 5 years of follow-up shows that clinically established restorative procedures (ACI-C, M-ACI, or OAT) are better suited to achieve good long-term clinical results with lower complication and failure rates than reparative procedures (microfracture), provided that the appropriate treatment indications including defect size of the respective restorative procedure are observed. In addition, ACI-C and M-ACI seem to be superior to OAT, especially in larger defects after longer follow-up periods.

## Supplemental Material

sj-pdf-1-car-10.1177_19476035221129571 – Supplemental material for Biological Reconstruction of Localized Full-Thickness Cartilage Defects of the Knee: A Systematic Review of Level 1 Studies with a Minimum Follow-Up of 5 Years Click here for additional data file. Supplemental material, sj-pdf-1-car-10.1177_19476035221129571 for Biological Reconstruction of Localized Full-Thickness Cartilage Defects of the Knee: A Systematic Review of Level 1 Studies with a Minimum Follow-Up of 5 Years by Peter Angele, Johannes Zellner, Steffen Schröter, Johannes Flechtenmacher, Jürgen Fritz and Philipp Niemeyer in CARTILAGE
