# Supplementary material for: Biological Reconstruction of Localized Full-Thickness Cartilage Defects of the Knee: A Systematic Review of Level 1 Studies with a Minimum Follow-Up of 5 Years
Source: Cartilage. 2022 Oct 15;13(4):5–18. doi: 10.1177/19476035221129571 (PMC9924981; doi:10.1177/19476035221129571)
Supplement: sj-pdf-1-car-10.1177_19476035221129571 – Supplemental material for Biological Reconstruction of Localized Full-Thickness Cartilage Defects of the Knee: A Systematic Review of Level 1 Studies with a Minimum Follow-Up of 5 Years [file sj-pdf-1-car-10.1177_19476035221129571.pdf]

Appendix 1: Search strategy for the systematic literature search in the Pubmed database and respective outputs

**Database provider/system**	PubMed	
**Date(s) of search**	17 November 2021	
**Period covered**	2011 to 2021	
**Filters**	article type (meta-analysis+systematic review+randomised controlled trial)+species (humans)+language (English+German)+publication date (2011 to 2021)	
**Name of safe search/file** (if applicable)	"PubMed Search"	
**#**	**Search Terms**	**Outputs**
1	cartilage repair[Title/Abstract]	114
2	cartilage defect[Title/Abstract]	41
3	cartilage restoration[Title/Abstract]	09
4	chondral defect[Title/Abstract]	10
5	osteochondral defect[Title/Abstract]	05
6	cartilage lesion[Title/Abstract]	16
7	chondral lesion[Title/Abstract]	09
8	autologous chondrocyte transplantation[Title/Abstract]	08
9	autologous chondrocyte implantation[Title/Abstract]	76
10	matrix-assisted autologous chondrocyte transplantation*	05

11	matrix-assisted autologous chondrocyte implantation*	02
12	microfracture[Title/Abstract]	102
13	marrow stimulation[Title/Abstract]	19
14	pridie drilling*	00
15	subchondral drilling*	07
16	abrasion arthroplasty*	06
17	mosaicplasty*	11
18	autologous osteochondral transplantation[Title/Abstract]	07
19	autologous matrix-induced chondrogenesis*	08
20	minced cartilage*	00
21	particulated cartilage*	02
22	knee joint replacement[Title/Abstract]	18
23	osteochondral repair[Title/Abstract]	00
24	allograft transplantation[Title/Abstract]	50
25	(osteochondral autograft transplantation[Title/Abstract]) OR (osteochondral autografting[Title/Abstract])) OR (osteochondral autograft[Title/Abstract])) OR (OC autograft[Title/Abstract])) OR (mosaicplasty[Title/Abstract])) OR (osteoarticular transfer system[Title/Abstract])) OR (osteochondral cylinder transplantation[Title/Abstract])) OR (osteochondral cylinder[Title/Abstract])) OR (autologous chondrocyte implantation[Title/Abstract])) OR (osteochondral allograft[Title/Abstract])) OR (OC allograft[Title/Abstract])) OR (microfracture[Title/Abstract])) AND (knee[Title/Abstract])	111

26	(matrix-induced autologous chondrocyte implantation[Title/Abstract]) AND (knee[Title/Abstract])	09
27	(Characterized chondrocyte implantation[Title/Abstract]) AND (knee[Title/Abstract])	02
28	(microfracture[Title/Abstract]) OR (autologous chondrocyte implantation[Title/Abstract])) OR (autologous transplantation[Title/Abstract])) OR (osteochondral autograft[Title/Abstract])) OR (osteochondral allograft[Title/Abstract])) OR (osteochondral transplantation[Title/Abstract])) OR (subchondral drilling[Title/Abstract])) OR (cartilage procedure[Title/Abstract])	242

*Not restricted to title/abstract.
